# Reply to Chambers, P. Comment on “Huțanu et al. Low Serum Vitamin D in COVID-19 Patients Is Not Related to Inflammatory Markers and Patients’ Outcomes—A Single-Center Experience and a Brief Review of the Literature. *Nutrients* 2022, *14*, 1998”

**DOI:** 10.3390/nu14163389

**Published:** 2022-08-18

**Authors:** Adina Huțanu, Anca Meda Georgescu, Septimiu Voidăzan, Akos Vince Andrejkovits, Valentina Negrea, Minodora Dobreanu

**Affiliations:** 1 Department of Laboratory Medicine, George Emil Palade University of Medicine, Pharmacy, Science, and Technology of Targu Mures, 540142 Targu Mures, Romania; 2 Department of Laboratory Medicine, Emergency Clinical County Hospital Targu Mures, 540136 Targu Mures, Romania; 3 Department of Infectious Diseases, George Emil Palade University of Medicine, Pharmacy, Science, and Technology of Targu Mures, 540142 Targu Mures, Romania; 4 Department of Epidemiology, George Emil Palade University of Medicine, Pharmacy, Science, and Technology of Targu Mures, 540136 Targu Mures, Romania; 5 Center for Advanced Medical and Pharmaceutical Research, George Emil Palade University of Medicine, Pharmacy, Science, and Technology of Targu Mures, 540136 Targu Mures, Romania

We thank Patrick Chambers for his interest [1] in our recently published paper on the vitamin D status in COVID-19 patients in the Romanian population [2]. Our study is a cross-sectional one, based solely on observational results and is not a randomized controlled one. We also stated that our patients did not receive a vitamin D treatment during the hospitalization, nor vitamin C (as vitamin C was mentioned in the commentary); we also did not state a dose recommendation for vitamin D supplementation neither in healthy nor in SARS-CoV-2-infected subjects.

The recommended thresholds for the vitamin D status, as well as the daily dose recommendation, both for bone homeostasis as well for extra-skeletal effects, including COVID-19, are still controversial, as recently reviewed by Bouillon R et al. [3]. Many factors drive these controversial results: different cut-off definitions, confounding factors, or different sampling moments. Although we are aware that the extra-skeletal effects of vitamin D are effective at higher serum levels, probably above 50 ng/mL, in our study we were not able to set this high threshold due to statistical reasons, since only a small number of subjects had reached this threshold.

A study published in JAMA by Murrai et al. that found no effect of high bolus-dose of vitamin D in COVID-19 patients used a baseline vitamin D level of 20 ng/mL [4]; however, the vitamin D supplementation reduced the acute respiratory infections, the daily dose is preferred upon intermittent dosage, and the lower the vitamin D levels, the greater the beneficial effects of vitamin D supplementation results [5].

In our study, there was no intention to assess the magnesium and calcium serum status; it was an evaluation of the vitamin D status in Romanian hospitalized COVID-19 patients. While the threshold values for blood levels are not yet universally accepted, they are not subject to on/off effects. We believe that evaluating the continuum of values in particular populations can still be meaningful.

Dr. Fauci’s personal preferences of supplements are undoubtedly informative but nevertheless cannot rank *per se* above the level of expert opinion.
